# Dutch Author Recognition Test

**DOI:** 10.5334/joc.95

**Published:** 2020-03-24

**Authors:** Marc Brysbaert, Longjiao Sui, Nicolas Dirix, Florian Hintz

**Affiliations:** 1 Department of Experimental Psychology, Ghent University, BE; 2 Max Planck Institute for Psycholinguistics, Nijmegen, NL

**Keywords:** reading, word recognition, language processing, author recognition

## Abstract

Book reading shows large individual variability and correlates with better language ability and more empathy. This makes reading exposure an interesting variable to study. Research in English suggests that an author recognition test is the most reliable objective assessment of reading frequency. In this article, we describe the efforts we made to build and test a Dutch author recognition test (DART for older participants and DART_R for younger participants). Our data show that the test is reliable and valid, both in the Netherlands and in Belgium (split-half reliability over .9 with university students, significant correlations with language abilities) and can be used with a young, non-university population. The test is free to use for research purposes.

## Individual differences in exposure to language

There is a large variability in the amount of language people are exposed to. This starts from a very early age on. Gilkerson et al. (2017) measured spoken language in over 300 families with children younger than three years. The average number of words said to the children by adults was about 12.5 thousand words per day. However, the standard deviation was over 5 thousand, meaning that the estimates ranged from less than 5 thousand words per day to more than 20 thousand words per day (see also Romeo et al., 2018).

Van Steelsel (2006) looked at differences in exposure to written language in 4–6 year olds. On the basis of interviews and cluster analysis, he found evidence for three types of families. The largest group (48%) mainly focused on activities that are seen as high priority for success in primary school, such as shared book reading, library visits, and learning rhymes (in songs and verses). The second family type (30%) focused on these activities to the same extent, but in addition engaged the children in reading and writing activities for daily usage (making shopping lists, reading advertising brochures, reading newspapers) and for personal development (reading books, magazines, use of personal computer, writing mails and postcards). The last family type (22%) did not engage the children much in any of these activities. Although there was a correlation with socioeconomic status and migration status, there were many exceptions. Only 18 of the 41 mothers with high education gave their children a rich home literacy environment (type 2), whereas 11 of the 20 mothers with low education belonged to types 1 or 2.

Individual differences in language exposure do not stop once children can read independently. A survey by Huysmans (2013) on 1,292 Dutch-speaking children indicated that 68% of 7-year olds read books daily against 10% once or twice a month at most. By the age of 15, the percentage of daily readers dropped to 21% while that of infrequent readers rose to 58%. The number of books read per month dropped from 3 for 7-year olds to 1.5 for 15-year olds. According to Kleijnen, Huysmans, and Elbers (2015), good school libraries can make a modest difference in the number of books read by children. With respect to adults, Swift and Ander (2017) on the basis of a Gallup survey in the US reported that 35% of Americans read 11 or more books in the previous year, 48% read 1–10 books, and 16% read no books.

Exposure to language, and in particular written language, correlates with language ability and success in school. For instance, van Steelsel (2006) found that, after controlling for socioeconomic and migration status, the home literacy environment predicted children’s vocabulary scores in first grade, and their general reading comprehension both in first and second grade. The effects of reading exposure on language processing and school performance have been confirmed in a large-scale meta-analysis (Mol & Bus, 2011) and tend to increase as individuals grow older, suggesting an upward spiral of causality in which print exposure and reading efficiency strengthen each other. Mol and Bus (2011) estimated that 12% of the variance in oral language skills is explained by print exposure in preschool and kindergarten children, 13%, in primary school, 19% in middle school, 30% in high school, and 34% in college and university.

## Measuring exposure to print

The existence of consistent individual differences with real-life implications has made print exposure an interesting variable for psychological research. However, rapidly the question arose about how best to measure it. Subjective estimates via questionnaires are an option, but usually are not very refined and may be open to bias, in particular to social desirability (reading is thought to be a good thing). This motivated Stanovich and West (1989) to develop an Author Recognition Test (ART) and a Magazine Recognition Test (MRT). In the ART, the names of 50 popular fiction authors were mixed with the names of 50 unknown individuals, and participants were asked to indicate which authors they knew. They were informed about the foils and told that they would be penalized if they selected any of the non-existing authors. The MRT was analogous to the ART and consisted of 50 titles of magazines and 50 made-up titles. To measure the usefulness of the ART and MRT, Stanovich and West (1989) correlated the test scores, together with the results of a reading questionnaire, to the outcome of a spelling test. Only the ART scores correlated significantly with the spelling scores (r = .46, N = 61). The usefulness of the ART was replicated in a second study with more dependent variables including the results of a word reading test and a reading comprehension test.

Acheson, Wells, and MacDonald (2008) updated the ART and MRT and reported similar findings as Stanovich and West (1989), including that ART was a better predictor than MRT and subjective estimates. Acheson et al.’s (2008) revised ART, which consists of 65 authors and 65 foils, has been used in most recent research. Another English version of ART was published by Mar and Rain (2015; see also Fong, Mullin, & Mar, 2013). These authors included 110 fiction authors, 50 non-fiction authors, and 40 foils. They too found that their ART correlated more with measures of verbal ability than self-reports of reading, and that the measure based on fiction authors did better than the measure based on non-fiction authors.

## Further evidence for the usefulness of the Author Recognition Test (ART)

Table 1 gives some correlational findings with ART scores in recent studies that included at least 85 participants (needed for 80% power to find correlations of .3). Early findings are summarized in Mol and Bus (2011). The correlations show that, apart from vocabulary knowledge, most correlations with ART are .3 or lower, in line with Mischel’s (1968) seminal observation that correlations with global person-related variables are of this size. This does not mean that the correlations are unimportant or unstable, but it means that we must measure them with enough precision.

**Table 1:** Correlations between scores on the Author Recognition Test (ART) and performance on other tests.

Study	Nparticipants	Dependent variable	Correlation

Dabrowska (2018)	90	Vocabulary size	.60
		Knowledge of word collocations	.50
		Education level	.47
		Language aptitude test	.45
		Grammar knowledge	.27
		Non-verbal IQ (Block patterns)	.09
Fong et al. (2013)*	328	Big Five Openness	.21
		Recognizing emotions in eyes	.17
		Big Five Extraversion	–.11
		Big Five Emotional stability	–.10
		Big Five Agreement	–.03
		Big Five Conscientiousness	–.02
James et al. (2018)	123	Vocabulary size	.45
		Pronunciation irregular words	.39
		Repetition nonwords	.28
		Survey reading habits	.25
		Phoneme reversal	.22
		Weekly reading time estimate	.19
		Stroop task	–.08
		Reading span	–.04
Mar & Rain (2015)*	340	Synonym knowledge	.32
	174	Reading comprehension	.26
	219	Sentence completion	.16
	227	Analogy knowledge	.13
Moore & Gordon (2015)	789	Word recognition (gaze duration)	–.38
Payne et al. (2012)	139	Vocabulary size	.62
		Sentence memory	.34
		Education	.30
		Reading comprehension	.26
		Reasoning	.20
		Reading span	.20
Samur et al. (2017)	366	Bluntness of feelings	–.28
	321	Bluntness of feelings	–.15
van Kuijk et al. (2018)	393	Education	.37
		Age	.37
		Recognizing emotions in eyes	.28
		Being absorbed by reading a text	.20
		Affective theory of mind	.18
		Cognitive theory of mind	.11
		Negative feelings	–.07
		Positive feelings	–.03

*Fiction authors only.

An interesting example is provided by the correlation between ART scores and performance on Theory of Mind tests. Theory of Mind (ToM) refers to the ability people have to attribute mental states to themselves and to others, and to understand that others have states that are different from their own. Understanding others’ mental states is a crucial skill that enables the complex social relationships characterizing human societies. A further distinction is sometimes made between affective ToM (the ability to detect and understand others’ emotions) and cognitive ToM (the inference and representation of others’ beliefs and intentions). Kidd and Castano (2013) argued that reading literary fiction increases ToM, pointing to two sources of evidence. The first was a positive correlation between ART scores and measures of ToM. The second was the finding that scores on ToM tests increased after reading a few relatively short texts of literary fiction. The latter has sparked much controversy, because its replicability was called into question. As a result, there have been several preregistered, high-power replication studies, including some by the original authors themselves (Kidd & Castano, 2019; Panero et al., 2016; Samur, Tops, & Koole, 2018; van Kuijk et al., 2018). Although the results have not been highly supportive for the claim that reading a few short literary texts increases scores on ToM tests, each and every study replicated the positive correlation between ART scores and performance on ToM tests (see also Mar, Oatley, Hirsh, dela Paz, & Peterson, 2006; Mar, Oatley, & Peterson, 2009, for earlier evidence relating the reading of fiction to abilities of empathy). Most authors have interpreted the positive correlation between ART and ToM performance as a causal effect from reading fiction to understanding others. However, Samur, Luminet, and Koole (2017) presented evidence that causality may be the other way round: People who have difficulty understanding others, are not keen on reading fiction. Needless to say, the correlation could also be due to a third factor related to both ART and ToM.

Not only the significant correlations with ART scores are important, also the non-significant correlations inform us about the interpretation of the measure (discriminant validity). Indeed, if ART scores are a true measure of exposure to print (in particular fiction), we ought not to be surprised by low correlations with non-verbal IQ or personality traits like agreement and conscientiousness (see Table 1). A further interesting observation is that the ART scores of scrabble-players are not higher than those of age-matched controls. Hargreaves, Pexman, Zdrazilova, and Sargious (2012) reported that although 57-year-old professional scrabble players know more words than age-matched controls, they do not have higher ART scores. Interesting was also that both groups had higher ART scores than university undergraduates, in line with van Kuijk et al.’s (2018) observation of a positive correlation between age and ART score (as shown in Table 1). We will return to this finding in Study 3.

## Non-English Author Recognition Tests

The status of English as lingua franca in psycholinguistic research means that it is more rewarding to develop resources English than for other languages. There are two reasons. First, there is more research done in English. So, there is more information available to build a good new test, and a newly developed measure is more likely to be used by colleagues. Second, it is harder to get research about non-English languages published in international journals, because editors and reviewers are more likely to question the usefulness of the measure, given that only part of the readers are familiar with the language.

As a result, it is difficult to find ARTs in other languages. Rodrigo, McQuillan, and Krashen (1996) compiled a Mexican Spanish ART consisting of 16 fiction writers and 9 foils. The test correlated .75 with a vocabulary test. Unfortunately, the finding does not look very safe, as the study was run with 19 participants only, the vocabulary test consisted of 16 words only, and the correlation between ART and the vocabulary test was higher than the reliability of ART (.61).

Chen and Fang (2015) published a Chinese ART for Taiwan, consisting of 75 real authors and 75 foils. They found that the test scores correlated .23 with vocabulary size, and .20 both with a reading comprehension test and a Chinese General Scholastic Ability Test. This was more than the correlations with self-ratings. Some further gain could be made by excluding secondary print knowledge from the ART (authors known by name but unlikely to be read by the participants).

Lee, Seong, Choi, & Lowder (2019) made a Korean ART, consisting of 40 popular authors and 40 foils. The test correlated r = .35 with a vocabulary test (60 items, multiple choice with four alternatives, N = 105 students), r = .39 with the accuracy data of a lexical decision experiment involving 120 words and 120 non-words, and r = .31 with a text comprehension test involving five texts and 20 comprehension questions. These correlations were higher than those with self-assessment of reading frequency.

Finally, a Dutch ART was proposed by Koopman (2015), consisting of 15 popular authors, 15 literary authors, and 12 foils. The scores correlated r = .26 with empathy for depression (N = 210 students) and r = .11 with empathy for grief. Koopman’s (2015) test was also used by Hartung, Burke, Hagoort, and Willems (2016), but seems to have been involved in one significant correlation only, namely the appreciation of the stories read (p < .05). Unfortunately, in neither article was information given about the reliability of the ART scores, so that it is difficult to evaluate the quality of the test.

In the sections below, we discuss a Dutch Author Recognition Test (DART) we developed independently of Koopman (2015).

## Compilation of the DART

The main challenge in building a good test is to find enough items of intermediate difficulty. Simple items known to nearly everyone and hard items known to virtually no-one are easy to find. What is more difficult to find, are the all-important items in-between, which drive the individual differences in test scores. Applied to a vocabulary size test, it is not so difficult to find words known to everyone and words known to very few; what is harder to track down, is words known to participants with a large vocabulary size in a particular sample but not to participants with a small vocabulary size. Yet, these are the most important for the test’s usefulness within that sample.

To build the DART on a firm basis, we started with a crowd-sourcing project. We obtained a list of almost 15 thousand fiction authors available at the library of Ghent (one of the larger cities in Flanders, the Dutch-speaking half of Belgium). To these, we added 7,600 foils. The foils were derived from lists of names that are unlikely to be known to the general public (e.g., participants in nonprofessional running contests, teachers from schools, people killed in World War I, etc.). Furthermore, we often recombined first names and family names, to further decrease the chances of including someone familiar. The language of the foils was matched to that of the authors (i.e., we had Dutch-sounding foils, French-sounding foils, English-sounding foils, and so on). Each participant in the crowdsourcing study received a random sample of 70 authors and 30 foils. Publicity was made via the university and newspapers. Data were analyzed after 20,000 individuals from Flanders (Belgium) and 5,000 individuals from the Netherlands had taken part (for more information, see Brysbaert, Mandera, & Keuleers, 2013).

An interesting finding was that fiction authors are not well-known. Even though the majority of people taking part in the crowdsourcing study were regular readers of 25–50 years, only 50 names were known to more than 90% of the participants. Fewer than 500 authors were known to at least half of the participants. Indeed, there were several complaints that the test was too difficult and did not measure the knowledge of “real” authors (very few participants recognized more than 10 authors out of the 70 presented).^1^

The outcome of the crowdsourcing study was that we had to limit the items to the 500 best-known authors (given that 18-year old students are likely to know even fewer fiction authors; Hargreaves et al., 2012). A further advantage was that we could more or less match the list for acquaintance in the Netherlands and Belgium. Just like for other languages shared by countries, there are considerable national differences in authors known.

Eventually, we selected 90 authors and 42 foils. The number of authors was rather high, because this is likely to increase the sensitivity and reliability of the test, and completing the test can be done rather rapidly (it only takes a few minutes). The number of non-author foils is lower, because few participants are expected to recognize more than 60 authors (in which case the number of no-responses [72] already exceeds that of yes-responses). The list is given in Appendix A.

The instructions were as follows (translated from Dutch): “This is a test to measure your knowledge of writers. You will get 132 names. Some of them are fiction writers. Please indicate the names of the authors you know. Be careful! Not all names are of writers and we will apply a correction for guessing if you select non-writer names as authors you claim to know. So, do not say yes if you do not know the author.”

## Evaluation of the DART

Given that the DART is built on the same principles as the English author recognition tests, we can expect to find similar correlations as those listed in Table 1, if the test reliably measures author knowledge and if it differentiates well. In the sections below we describe the outcome of five studies, three in Belgium and two in the Netherlands.

### Study 1

The first study is described in detail in Vander Beken and Brysbaert (2018). It involved memory for texts studied in the native language and in English as second language. Undergraduates from Ghent University studied short expository texts for seven minutes in Dutch or in English. Subsequently, they had to recall as much as possible from the text or answer yes/no recognition questions. A total of 195 students took part. They all completed the DART and a series of other tests, including vocabulary tests and a non-verbal IQ test.

The DART-score was calculated as the percentage author names indicated minus the percentage non-authors wrongly selected. So, a participant who indicated they knew 30 of the 90 authors and wrongly said that 1 of the 42 foils was an author they knew, would get a score of 30/90–1/42 = .31 or 31%. The average score was 24% (SD = 14), in line with the observation that fiction authors are not very well known to the general public. The percentage of authors selected was 35%. The percentage of foils selected was 11%, indicating that the correction for guessing was needed. Internal item consistency of the test was .97, measured with the split-half reliability between the first and the second half of the test and attenuated for length.

Table 2 shows the correlations with the other tests administered. For the correct interpretation of these correlations, it is good to know that some of the tests had lower reliability than aimed for. In one case, this was because the test was too easy (LexTale Dutch, which is a test for Dutch as a second language). In another case this was because the test had not yet been optimized (the Dutch vocabulary test with multiple choice items, an improved version of which was eventually published in Vander Beken, Woumans, & Brysbaert, 2018, with a reliability of .84; see also Study 5). Finally, the Raven Matrices test had been administered in a suboptimal way (the items were shown for a fixed time to groups of participants, rather than letting everyone work at their own pace). It can be expected that the correlations would be higher for improved test versions/applications (see Table 6).

**Table 2:** Correlations DART with other tests (N = 195 students). These correlations can be compared to those obtained in English, as shown in Table 1. For each test, the reliability is given, as for some tests it was suboptimal. Source: Vander Beken & Brysbaert, 2018.

Test	Correlation with DART	Reliability test

Yes/No Vocabulary test Dutch (Lemhöfer & Broersma, 2012)	.05	.63
Vocabulary test Dutch (multiple choice)	.42	.66
Dutch spelling test	.27	.87
Yes/No Vocabulary test English (Lemhöfer & Broersma, 2012)	.30	.90
Non-verbal IQ (Raven)	.06	.46

Correlations are significant at p < .05 when larger than .15 and at p < .01 when larger than .19.

### Study 2

The second study addressed the question whether introvert people read more than extravert people (Vandevyvere, 2017). Frequent reading is part of Eysenck’s definition of introversion, as can be seen in the following book extract: “The typical introvert is a quiet, retiring sort of person, introspective, *fond of books rather than people*; he is reserved and distant except to intimate friends” (Eysenck & Rachman, 1965, p. 19, emphasis added). Book-reading also seems to be part of lay-people’s understanding of introversion. When first-year students psychology are asked about frequent book reading, they see this as a typical characteristic of introverts, as strongly as being silent in the presence of unfamiliar people (Vandevyvere, 2017).

Contrary to Eysenck’s definition and general expectation, book reading is not highly correlated with introversion. In Table 1 we saw the data of Fong et al.’s (2013), who obtained a correlation of –.11 with extraversion, which is in the right direction but low and about half the correlation with the Big Five personality trait “Openness to experience”. Table 3 shows that this finding is quite consistent across studies. The correlation seems to be slightly higher for direct diary recordings than for estimates of reading frequency based on self-assessment or ART.

**Table 3:** Correlations between reading frequency on the one hand and extraversion and openness to experience on the other hand, reported in various studies.

Study	Measure of reading	Nparts	Corr. with extraversion	Corr. with openness

Finn (1997)	Diary recordings	219	–.23	.27
Fong et al. (2013)	ART-Fiction	328	–.11	.21
Kraaykamp & van Eijck (2005)	Likert scale	3156	.02	.16
Mar et al. (2009)	ART-Fiction	252	–.04	.22
McManus & Furnham (2006)	Likert scale	1071	–.05	.26
Oerlemans & Bakker (2014)	Diary recordings	1364	–.15	NA

There may be two reasons why introverts are not more likely to read than extraverts. First, as we saw above, it has been argued that fiction reading helps understanding others, which may interest extraverts as much as introverts (Kidd & Castano, 2019; Mar et al., 2006, 2009). Second, there are different types of books. So, it could be that extraverts read other books than introverts. Although this is a sensible hypothesis, it has not received much empirical support. Rentfrow, Goldberg, and Zilca (2011) argued that leisure activities (listening to music, watching TV and movies, reading books) consist of five dimensions: Communal (romance and entertainment), aesthetic (classical music and arts), dark (punk music, heavy metal, and horror books/films), thrilling (action books/films, thrillers), and cerebral (non-fiction). Personality differences correlated with preferences on these dimensions. However, specific for extraversion, there was no clear line throughout the findings and the correlations went opposite to those in Table 3 (i.e., there was a positive correlation between entertainment use and extraversion). Other authors who looked at differences in reading preferences between introverts and extraverts (Fong et al., 2013; Lau & Cheung, 1988; Schutte & Malouff, 2004) also failed to find strong, consistent effects of extraversion. So, chances seem low that a much better correlation will be found between reading and introversion, when reading is limited to a particular genre.

Vandevyvere (2017) tested to what extent the same pattern of findings would be found with the DART. She made use of a Dutch translation of the Big Five Inventory (John, Donahue, & Kentle, 1991) and verified that the translation was as reliable as the original version. The questionnaire was presented via the internet to a community sample of 263 participants, who also completed the DART, and some Likert scales about their reading frequency. Average score on the DART was 27% (SD = 16). Reliability of the DART was .95 as measured with Cronbach’s alpha on the author items.^2^

Table 4 shows the correlations between the DART scores and the other variables measured. These are comparable to what has been found in English. Further interesting is that the correlations between openness/extraversion and ART were higher than those with the subjective estimates. For instance, the correlation between openness and the answers to the question “how many books did you read in the past year” was .14 (compared to .19 for ART). The correlation between extraversion and the answer to the question was –.05 (compared to –.09 for ART).

**Table 4:** Correlations of test results and question answers with the DART scores (N = 263). Source: Vandevyvere (2017).

Measure	Correlation

BFI – Openness	.19
BFI – Conscientiousness	–.11
BFI – Extraversion	–.09
BFI – Agreeableness	–.14
BFI – Neuroticism	–.05
“How many books did you read in the last year?”	.46
“How many newspapers did you read the last month?”	.10
“How many journals/magazines did you read the last month?”	.07
“How much do you read relative to other people?”	.44

Correlations are significant at p < .05 when larger than .13 and at p < .01 when larger than .16.

### Study 3

The third study was run in Nijmegen (The Netherlands) at the Max Planck Institute for Psycholinguistics in the spring of 2018 and compared performance of 85 participants on a series of tests. About half of the participants (N = 41) were younger than 30 years (mean age = 23), the others were older than 60 years. The groups were matched on years of education (all had completed or were studying for a university degree). Reliability of the DART was .98, as measured with the split-half correlation between the first and the second half, attenuated for length. On average, participants responded yes to 43% of the authors and 2% of the foils.

As reported by Hargreaves et al. (2012) and van Kuijk et al. (2018), there was a positive correlation between age and DART scores (r = .73; Table 5). The old group performed much better (M = 59; SD = 16.8) than the young group (M = 24; SD = 14.4). Two elements seem to be involved. First, older people have had more time to read books than younger people. As a result, they know more author names, just like they know more words (Brysbaert, Stevens, Mandera, & Keuleers, 2016). At some point in very old age, knowledge is likely to decrease again when memory starts to suffer, as has indeed been reported by Payne, Gao, Noh, Anderson, and Stine-Morrow (2012) for ART scores.

**Table 5:** Correlations DART with other tests (N = 85). These correlations can be compared to those obtained in English, as shown in Table 1. If available, the reliability of the test is given. Source: Rosenbaum (2018).

Test	Correlation with DART	Reliability test

Age of the participants	.73	NA
Receptive vocabulary size	.28	NA
Non-verbal response time	.34	NA
Phrase production time	.29	.90
Sentence production time	.37	.79

Correlations are significant at p < .05 when larger than .22 and at p < .01 when larger than .28.

A second element, however, was that the items in the DART may have favored older participants over youngers ones. Because of the initial crowd-sourcing study, estimates of author knowledge were mainly based on an older audience. Indeed, looking at the items much better known by older participants than younger, we couldn’t help but notice that many of these items were authors popular at the end of the 20^th^ century (i.e., before current-day undergraduate students were born). We will return to this issue in Study 5.

Table 5 further shows the correlations with other test results. It also includes information about the reliabilities of the tests, if available. We tried out four tests. The first assessed participants’ receptive vocabulary size (Hintz et al., 2018). Participants responded to words of varying difficulty by indicating whether they knew the word or not. Using a staircase procedure, the test adapted to the performance level of each participant. The participant’s score was the most difficult level for which they indicated they knew the words. The second measure was a factor score derived from a simple and a complex non-verbal auditory processing speed task. In the simple speed task, participants were instructed to push a button as quickly as possible upon hearing a 550 Hz sine tone. In the complex speed task, participants heard a low (300 Hz) or high (800 Hz) sine tone and were instructed to push the button associated with each tone as quickly as possible. Performance indicator in both tasks was the average response time (only correct trials in the complex speed task). Response times more than 2.5 SD away from a participant’s mean were considered outliers and removed. The third and the forth test addressed phrase and sentence production abilities, respectively, and were administered as two parts of the same test (Rosenbaum, 2018). In the phrase production part, participants produced phrases of increasing syntactic complexity: Ranging from simple noun phrases (“book”, “belt”), concatenations (“book and belt”) to simple and complex adjective phrases (“yellow belt”, “two blue books”). The objects were visually simple and known to all participants. Performance indicator was the duration of the (correct) phrase production, averaged over the different kinds of phrases. In the sentence production part, participants produced transitive sentences in active and passive voice using the paradigm and stimuli described in Menenti et al. (2011). As in the phrase production part, performance indicator was the average duration of the correctly produced utterances. In the test session, participants first performed the phrase and sentence production tests, followed by the receptive vocabulary test, the speed tasks and the DART. They subsequently did the phrase and sentence production tasks again. The reliability reported in Table 5 therefore refers to the correlation between performances in both runs (i.e. test-retest reliability).

As in the English studies (e.g. James et al., 2018) and in Study 1, we observed a positive correlation between vocabulary size and author knowledge. Interestingly, performance on the non-verbal processing speed tasks also correlated positively with performance on the DART. This correlation is most likely driven by the older participant group as these participants displayed larger knowledge of authors while performing more slowly (i.e. larger response times) on the processing speed tasks as compared to the younger participants. A similar explanation is likely to apply to the positive correlation between phrase and sentence production performance and performance on the DART: Older participants who tended to speak more slowly (i.e. longer) than the younger participants displayed better author knowledge than their younger peers.

### Study 4

The fourth study addressed the question to what extent the DART is useful for a less educated, young population. Participants were 72 students from vocational higher education (mean age = 20 years, range 18–25). They responded yes to 7% of the authors and 1% of the foils, giving an average DART score of 5.9 (SD = 4.4). Reliability of the test for this group was .71, as measured with the split-half correlation between the first and the second half of the test, attenuated for length.

Table 6 gives the correlations with four other tests the participants completed. As before, we include information about the reliability of the tests, if available. As in Study 3, the first test assessed receptive vocabulary (Hintz et al., 2018). The second (custom-made) test assessed participants’ spelling abilities for words whose spelling has been shown to be difficult for adult Dutch speakers (e.g., use of the graphemes ei vs. ij, consonant doubling in plurals, use of diaresis). The third test was a grammatical judgment task. Participants listened to sentences and were instructed to judge whether the sentences followed the Dutch grammar rules. The sentences featured five grammatical categories, which adult native speakers of Dutch often find difficult to use correctly: personal pronouns (“ze” vs. “hun” and “ik” vs. “mij”), comparatives “als” vs. “dan”, relative pronouns “die” vs. “dat”, and participle formation of complex verbs, such as “stofzuigen” (to vacuum). The fourth test was Raven’s advanced progressive matrices. Participants had 20 minutes to complete 36 experimental items, which increased in difficulty. The correlations between these four tests and DART largely replicate previous results from English. The relatively low correlation with the grammar test was surprising, but is likely to be due to the low reliability of the test.

**Table 6:** Correlations DART with other tests for a sample of non-university participants (N = 72). Source: Unpublished data collected by Hintz, Dijkhuis, van ‘t Hoff, McQueen & Meyer.

Test	Correlation with DART	Reliability test

Knowledge words	.33	*NA*
Spelling test	.24	.73
Grammar test	.08	.31
Non-verbal IQ (Raven)	.20	.79

Correlations are significant at p < .05 when larger than .24 and at p < .01 when larger than .31.

### Study 5

As we mentioned in Study 3, we noticed that some authors popular a few decades ago were not known to the younger participants. Even though reliability of the DART is as good as it can get, the scores of young participants were rather low and items not known to them are redundant. The scores are likely to decrease further, as the test gets older. For that reason, we looked which authors of the test were not selected more than the foils by the young participants of our studies. These were: Toni Morrison, Sue Grafton, Raymond Chandler, Marianne Frederiksson, Hubert Lampo, Stefan Zweig, Dante Alighieri, Uwe Tellkamp, Donna Tartt, Ruth Rendell, Ray Bradbury, Michael Connelly, Mario Vargas Llosa, John Le Carré, P.F. Thomése, Fay Weldon, Joris Van Casteren, Manon Uphoff, Per Olov Enquist, Georges Simenon, Milan Kundera, Daniel Mason, Henning Mankell, Roberto Bolano, and David Grossman. They were replaced by who more likely to be read by Dutch-speaking children and young adults: Haruki Murakami, Jeff Kinney, Esther Verhoef, Lucinda Riley, Carry Slee, Santa Montefiore, Manon Sikkel, John Green, Stephenie Meyer, Jostein Gaarder, Jussi Adler-Olsen, Paulo Coelho,^3^ Francine Oomen, Michel Houellebecq, Paul van Loon, Suzanne Vermeer, Elena Ferrante, Sarah J Maas, Griet Op de Beeck, Liz Pichon, Suzanne Collins, E.L. James, John Flanagan, Rick Riordan, and Tonke Dragt. The revised version is shown in Appendix B.

The new questionnaire (DART_R) was used in a study with 62 participants, all students from Ghent University (mainly undergraduate students). Average performance on the test (after correction for false alarms) was 34% (SD = 14). False alarm rate was 2%. Performance was 7–10% higher than in the first two studies. Reliability was .95 when measured with Cronbach’s alpha (author names only) and .92 when measured on the basis of the split-half correlation attenuated for length between the first and the second half of the test.

Participants also took part in five validation tests (Table 7). The first test was the Lextale vocabulary test of Lemhöfer & Broersma (2012) as in Study 1, even though this test has a ceiling effect for native speakers. The second test was the multiple choice vocabulary test published by Vander Beken et al. (2018; see also Study 1). The third test was a spelling test from GL&SCHR, a test battery for students with dyslexia (De Pessemier & Andries, 2009), consisting of 30 words that were dictated. The fourth test was a short version of Cattell’s Culture Fair Intelligence (CFT20; Weiß, 2006). It consisted of 12 matrices and was included to obtain an estimate of fluid intelligence. Finally, the last test consisted of reading 12 short texts of some 150 words. For each text, the reading rate was measured.

**Table 7:** Correlations DART_R with other tests (N = 62 students).

Test	Correlation with DART_R	Reliability test

Yes/No Vocabulary test Dutch (Lemhöfer & Broersma, 2012)	.26	.61
Vocabulary test Dutch (multiple choice)	.64	.87
Dutch spelling test	.34	.60
Non-verbal IQ (CFT20)	–.10	.51
Reading rate	.38	.96

Correlations are significant at p < .05 when larger than .26 and at p < .01 when larger than .33.

As in Study 1, the new DART_R correlates well with the vocabulary tests. Because the multiple choice test has been improved relative to Study 1 (reliability = .87), the correlation with DART_R has increased. DART_R also correlates well with reading speed (M = 228 words per minute; SD = 55). The correlation between DART_R and reading speed (r = .38) is higher than the correlation between the scores on the multiple choice vocabulary test and reading rate (r = .29). Finally, DART_R does not correlate with the test of non-verbal, fluid intelligence, although it must be noted that the test used was too short to give refined estimates for the student sample we tested (reliability of only .51).

A further addition we tried out is a largely overlooked element in Stanovich and West (1989). These authors not only pioneered the ART and MRT, but also asked participants to name their two favorite authors. This variable correlated almost as much with the criterion variables as the ART did in Stanovich and West (1989). So, we examined whether the print exposure measure could be improved if at the end of the DART_R we asked the participants “Do you have favorite authors not included in the list?” and gave them three entries for answers. Against our expectations, when we included the variable in regression analyses (operationalized in different ways) it failed to significantly improve the predictions for the validation tasks. Still, researchers may want to retain the element, as it can provide them with interesting names for future adaptations of DART_R.

## Discussion

We presented and evaluated the Dutch Author Recognition Test (DART and DART_R). The most important finding is that we managed to find a list of authors and foils that reliably measure knowledge of fiction authors in The Netherlands and Belgium. Reliability is above .9 for participants with university education and above .7 for participants without such education. The difference between both groups reminds us that reliability is sample dependent. A test made for students is likely to have a lower reliability for non-students, if most non-students have low scores (as happened in Study 4). Similarly, a test with good reliability in the population at large may have low reliability in a student population if most students score very well (due to range restriction). This is what happened for a few of the tests we tried out (e.g., LexTALE, the spelling test and CFT20 in Study 5).

We started with the DART and saw that it was well suited for adult and elderly participants, but tended to give low scores for students in high school and undergraduates. To improve the latter, we replaced some of the older author names that were not known to young participants. This increased the average scores for students in Study 5, although it did not increase the reliability of the test for them (which was already high). We recommend using the DART_R version with young participants and the DART version for research with older participants.

Looking at the correlations with participant variables and other test scores, we have good reasons to assume that the DART and DART_R are of the same quality as the English ARTs used for research (Acheson et al., 2008; Mar & Rain, 2015). Reliabilities are comparable and test results correlate well with measures of language ability, reading speed and the Big Five personality dimension Openness. The DART scores do not correlate much with the other Big Five personality dimensions or with fluid intelligence.

It is true that the correlations of DART with other variables are often low, also for variables that are assumed to be related.^4^ This is the case for the English findings as well (Tables 1 and 3). Two elements are involved. First, the typical correlation between person characteristics and behavioral variables is r = .2 (Gignac & Szodorai, 2016). The same is true when one tries to predict text difficulty on the basis of text characteristics: the majority of variables correlate .2–.3 and add but a small percentage of variance explained (e.g., Crossley, Skalicky, Dascalu, McNamara, & Kyle, 2017). To some extent, this is to be expected. If the correlation between DART and vocabulary size were much higher than r = .6, we would have to conclude that vocabulary size is entirely dependent on fiction reading or that fiction reading entirely depends on the participant’s vocabulary size. Similarly, if the correlation between ART scores and the Big Five dimension Openness were .8, this would suggest that Openness almost entirely consists of reading fiction books. So, for many predictors correlations with ART are bound to be in the order of r = .2 to .4; fiction book reading only explains some of the variance.

A second reason why correlations with DART were sometimes lower than expected in our studies is that the quality of the predicted variables was lower than we had hoped for. This was partly because we were trying out new tests that were not yet fully optimized, but partly also because tests made for a wider segment of the population often do not work well with students, due to range restriction. A way to assess the maximum possible correlation corrected for the unreliability of the measures is to use the equation:

CorrectedCorrelation = \frac{{ObservedCorrelation}}{{\sqrt {reliabilit{y_{test1}}*reliabilit{y_{test2}}} }}

For instance, the corrected correlation between the DART and the multiple choice vocabulary test in Study 1 is .42{\rm{/}}\sqrt {.97*.66} = .53. For Study 5 it is: .64{\rm{/}}\sqrt {.92*.87} = .72.

## Data Accessibility Statement

The DART and DART_R can be used freely for research purposes under the Attribution-NonCommercial-ShareAlike (CC BY-NC-SA) scheme of Creative Commons. The tests are given in the Appendices. They are also available as Excel files on https://osf.io/u4vhs/. The osf website further includes the raw DART data of Studies 1 and 3 – 5. Those of Study 2 unfortunately are no longer available.

## Additional Files

The additional files for this article can be found as follows:

10.5334/joc.95.s1 Appendix A. DART Excel versie.

10.5334/joc.95.s2 Appendix B. DART_R Excel versie.

## Footnotes

In hindsight, these numbers could have been expected. Even a person reading one book per week, has only read 2,080 books after 40 years, many of which will be from the same authors.

Unfortunately, no other measures of reliability were reported and the raw data are no longer available. Given that there are two types of items (authors and non-authors) and that a corrected score is used, arguably the split-half correlation is the most useful. As will be shown below, however, there is little difference between the two measures when both are calculated on the same dataset.

Initially it was Geronimo Stilton, but this is a character rather than an author. Another typo we noticed at a very late stage was the author Susan Smith. This should be S.E. Smith or Susan Smit. Given that the latter is much more likely to be known to Dutch-speaking readers, we recommend using her name. The changes have been made in the appendices.

We thank one of the reviewers for pointing this out to us.
